# Systematic analysis of approaches used in cardiac arrest trials to inform relatives about trial enrolment of non-surviving patients

**DOI:** 10.1136/emermed-2023-213648

**Published:** 2024-05-10

**Authors:** Helen Pocock, Abigail Dove, Laura Pointeer, Keith Couper, Gavin D Perkins

**Affiliations:** 1 University of Warwick Medical School, Coventry, UK; 2 South Central Ambulance Service NHS Foundation Trust, Bicester, Oxfordshire, UK; 3 Critical Care Unit, University Hospitals Birmingham NHS Foundation Trust, Birmingham, UK

**Keywords:** Out-of-Hospital Cardiac Arrest, heart arrest, Randomized Controlled Trial, research design, research

## Abstract

**Background:**

The recruitment of patients to emergency research studies without the requirement for prior informed consent has furthered the conduct of randomised studies in cardiac arrest. Frameworks enabling this vary around the world depending on local legal or ethical requirements. When an enrolled patient does not survive, researchers may take one of three approaches to inform relatives of their enrolment: a direct (active) approach, providing information indirectly (passively) and inviting relatives to seek further information if they choose, or providing no information about the trial (no attempt). Previous studies have described experiences of US researchers’ active approach but there is little known about approaches elsewhere.

We aimed to conduct a structured investigation of methods used in cardiac arrest trials to provide information about trial enrolment to relatives of non-surviving patients.

**Methods:**

We systematically searched trial registries to identify randomised clinical trials that recruited cardiac arrest patients. Trials were eligible for inclusion if they recruited adults during cardiac arrest (or within 1 hour of return of spontaneous circulation) between 2010 and 2022 (in the decade prior to study conception). We extracted data from trial registries and, where relevant, published papers and protocols. Investigators were contacted and asked to describe the style, rationale and timing of approach to relatives of non-surviving patients. We present descriptive statistics.

**Results:**

Our trial registry search identified 710 unique trials, of which 108 were eligible for inclusion. We obtained information from investigators for 64 (62%) trials. Approximately equal numbers of trials attempted to actively inform relatives of non-survivors (n=28 (44% (95% CI; 31% to 57%))), or made no attempt (n=25 (39% (95% CI; 27% to 52%))). The remaining studies provided general information about the trial to relatives but did not actively inform them (n=11 (17% (95% CI; 8% to 29%))).

**Conclusions:**

There is wide variability in the approach taken to informing relatives of non-surviving patients enrolled in cardiac arrest randomised clinical trials.

**WHAT IS ALREADY KNOWN ON THIS TOPIC**

- In the UK cardiac arrest researchers do not routinely attempt to actively inform relatives of non-surviving patients of their enrolment in a trial. This contrasts with the US approach, which is mandated in law, but elsewhere other factors may determine the approach taken.

**WHAT THIS STUDY ADD**

- This study systematically investigates the methods by which information is provided to relatives by researchers. We report the various approaches, influences and issues in 62% of the cardiac arrest trials registered around the world in the period 2010–2022. We found wide variability in practice.

**HOW THIS STUDY MIGHT AFFECT RESEARCH PRACTICE OR POLICY**

- We suggest further research is needed into the relatives’ perspective to inform a best practice recommendation.

## Introduction

### Background

 The recruitment of patients to emergency research studies without the requirement for prior informed consent has extended the possibility of improving care through research to this previously neglected patient group. The legal and ethical frameworks governing this, and associated practices vary around the world.

Similarly, the approach to notification of relatives when a participant in the trial does not survive also varies. We are aware for instance, of major differences in approach between UK and US settings. To date, it has not been common practice in UK cardiac arrest trials to actively inform the relatives of non-surviving patients of their inclusion in research. This has been based on concerns surrounding the potential burdens to the recipients of this information and the practicalities of delivering such communications. Such an approach has been supported by patient and public advisors.^1^,^3^ Previously, either no approach or a ‘passive’ approach has been made to relatives to avoid or minimise the introduction of additional emotional burden at a time of great distress.^1 2 4^ A passive approach is one where general trial information is targeted to locations where relatives are likely to come across it. This information includes an invitation to seek further information should they wish. It places the choice of whether or not to find out more with the relatives. But this is in direct contrast to practice elsewhere.

In the USA the approach is mandated in law. Patients without capacity may be enrolled in drug and device research under the exception from informed consent regulations or the waiver of informed consent regulations for other types of study.^5 6^ The former requires prior community consultation, whereby information is provided and opinions sought in advance of the research to inform the ethics board and to demonstrate the requisite respect for community members. In both types of study, if the patient dies before a legally authorised person can be contacted, the enrolment must be disclosed to them if possible.^7^ Researchers are required to attempt to notify relatives and this is usually communicated via letter.^8 9^ Difficulties with reliably obtaining valid contact information have been identified but studies report between 83% and 91% success in acquiring correct details and sending such letters.^8^,^10^ Despite some difficulties, the information contained in the letters appears to have been acceptable to recipients since consent for data use was refused in fewer than 1% of cases.^9 10^

During our recent cardiac arrest trial our patient and public partners cautiously stated their preference for actively informing the relatives of non-surviving patients.^11^ With no UK precedent we sought the experience of researchers elsewhere. There is a need for a structured investigation to describe the different approaches taken by researchers across the world to inform the relatives of non-survivors which may guide others and inform practice. The primary objective was to determine the approach taken to informing the relatives of non-survivors enrolled in cardiac arrest trials recently published, registered or currently being conducted. The secondary objectives were to describe the methods used, to establish what influenced the selection of the approach and whether researchers experienced any specific issues.

## Methods

### Study design and setting

We conducted a systematic analysis of the practices of researchers in cardiac arrest trials using an online data collection tool (see online supplemental materials).

The project was carried out according to the Helsinki declaration and principles of Good Clinical Practice. We report our study in accordance with the Strengthening the Reporting of Observational Studies in Epidemiology checklist for observational studies.^12^

### Selection of participants

To identify researchers, we searched international trial databases containing details of trials meeting WHO and International Committee of Medical Journal Editors registration criteria and issued with a registration number whether planned, underway or completed.^13 14^ Specifically, in line with current best-practice guidance, we searched ClinicalTrials.gov, a US-hosted resource including studies from 221 countries, and the WHO International Clinical Trials Registry Platform meta-register, which contains trial records from 18 registries from across the world.^15^

We contacted the lead investigators of studies fulfilling the following eligibility criteria:

Inclusion criteria

- Randomised or quasi-randomised clinical trials registered, currently being undertaken, or published since 1 January 2010.
- Effectiveness or efficacy trial of an intervention delivered intra-arrest or within 1 hour of return of spontaneous circulation.
- Trial includes adult patients only (as defined by local legal or clinical definition).
- Patients enrolled without prior informed consent.

Exclusion criteria

- Unable to determine eligibility status from registry record and trial publication/protocol not available in English.

We searched trial registries on 05 October 2022 for interventional studies using the term ‘cardiac arrest’. To capture studies reporting from 2010, the search included studies first posted from 01 January 2007. Three years of balanced sensitivity and specificity for identification of studies within resource limitations. Screening for relevance was conducted by title by a single author (HP). Eligibility was determined by a full registry entry review and 10% was checked by a second author (KC). Discrepancies were resolved by a third author (GDP).

### Data collection

We sought information regarding approaching the relatives of non-surviving patients from the registry record or from published papers and protocols. By searching using the study title or acronym from the registry, or keywords from that title, we identified publications and protocols. If required, publications were searched on ResearchGate.^16^ If this information was not identified in these sources, we contacted researchers directly. Researchers’ contact information was obtained either from the registry record or from any other publications they had coauthored. We sent emails to individual recipients to minimise the chance of them being perceived as spam, and customised with their names and titles to make the invitation more personal.^17^ Separate emails were sent for each trial and researchers were invited to respond separately to each of these. If we received no response after 7 days, the invitation email was sent again.

We constructed the data collection tool using Qualtrics XM (2005–2022, Provo, Utah, USA).^18^ The content of the tool was informed by experienced critical care researchers and items were carefully worded for ease of interpretation and international applicability. To maximise online readability and ease of response each item was presented on a new page with a consistent spatial arrangement, colour and font.^19 20^ We applied skip logic so that respondents viewed only relevant questions depending on their previous responses.^18^ To minimise the cognitive load on respondents, the number of items was reduced to a minimum and no responses were forced.^17 21^ Clinical sensibility testing of questions was conducted among experienced cardiac arrest researchers to establish face validity.^19^ The tool was piloted for comprehension and performance, such as ensuring that skip logic and multiple response options were functional. A participant information sheet was embedded, and consent was sought prior to accessing the tool. We conducted data collection from December 2022 to January 2023 and de-identified all responses (by respondent) prior to analysis.

### Data analysis

We summarised and presented quantitative survey responses using descriptive statistics. Qualitative data responses were subject to summative content analysis whereby keywords were identified to interpret the contextual meaning of the content.^22^ We compared characteristics of responder trials and non-responder trials using a χ^2^ test. Data analysis was performed by a single researcher (HP) and checked by a second (KC).

### Patient and public involvement

Discussion with patient and public partners inspired this study, but as this was an investigation of researcher practice, they were not involved in the study design.

## Results

### Characteristics of study participants

**Figure 1:**
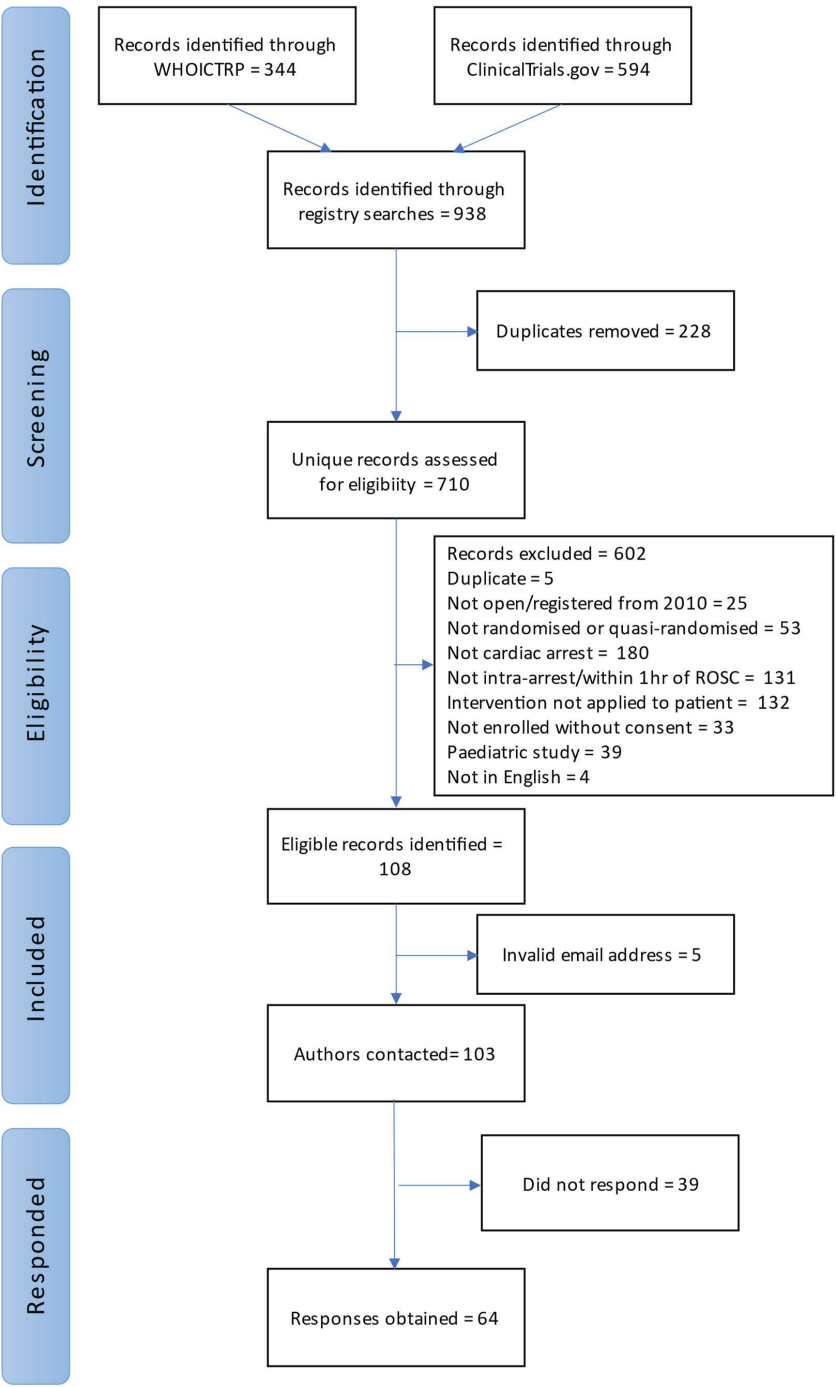
Study flow diagram. ICTRP, International Clinical Trials Registry Platform.

We identified 710 unique records through registry searches (figure 1). After a review of trial records, we retained 108 for analysis. A summary of the study characteristics (population, intervention, comparator, primary outcome) is provided in the online supplemental material.

Since it was not possible to obtain the required information from the registries, publications or protocols, invitations to participate in the study were sent out for all studies (n=108). Five email addresses were returned as unknown and alternative addresses could not be identified. Information relating to 64 studies was provided by researchers (62% response rate). There was one ‘duplicate’ study (entered with a different title in different registries) but responses were not conflicting so the second response was removed from the data set.

Most trials that responded were set in the out-of-hospital setting (n=48, 75%), in Europe (n=40, 63%) and were not drug trials (n=46, 72%) (table 1). We observed some differences in trial characteristics between responding trials and non-responding in relation to cardiac arrest setting and location of recruitment.

**Table 1:** Characteristics of responding and non-responding studies

		Responders n=64 (62.1%)	Non-responders n=39 (37.9%)	P value (Pearson χ^2^)
Location of cardiac arrest	Out-of-hospital	48 (75)	34 (87)	p=0.05*
	In-hospital	9 (14)	0 (0)	
	Both	7 (11)	5 (13)	
Continent of recruitment	Europe	40 (63)	20 (51)	p=0.016*
	North America	15 (23)	9 (23)	
	Australasia	5 (8)	0 (0)	
	Asia	4 (6)	10 (26)	
Type of intervention	Drug	18 (28)	13 (33)	p=0.576
	Non-drug	46 (72)	26 (67)	
Year study opened	Pre-2015	22 (34)	17 (44)	p=0.636
	2015–2019	22 (34)	11 (28)	
	2020 onwards	20 (31)	11 (28)	

* Significant at p≤0.05

### Main results

**Figure 2:**
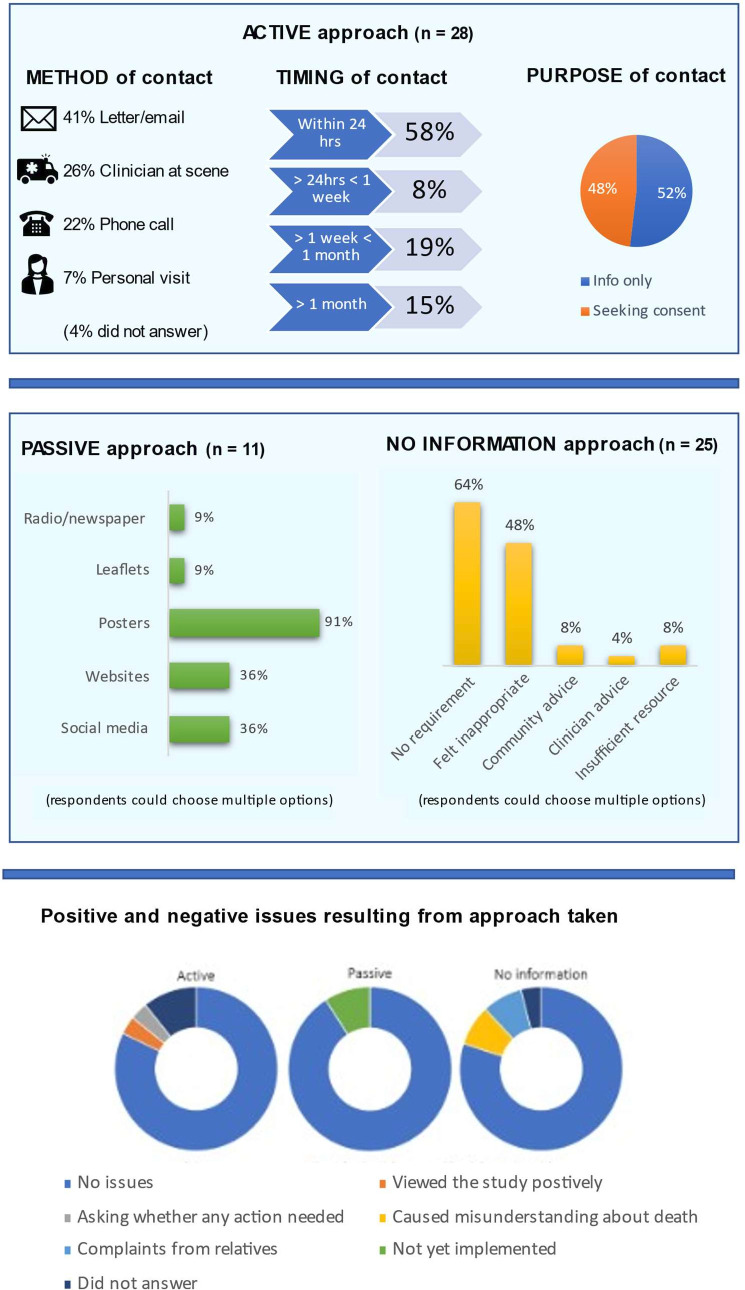
Type, method and rationale of approach taken with associated resulting issues.

In 28 studies (44% (95% CI; 31% to 57%)), researchers took an active approach to informing relatives of non-surviving patients about study enrolment (figure 2). In an approximately equal number of studies (n=25 (39% (95% CI; 27% to 52%))) no information was provided to relatives. A passive approach to information provision was taken in 11 studies (17% (95% CI; 8% to 29%)).

**Figure 3:**
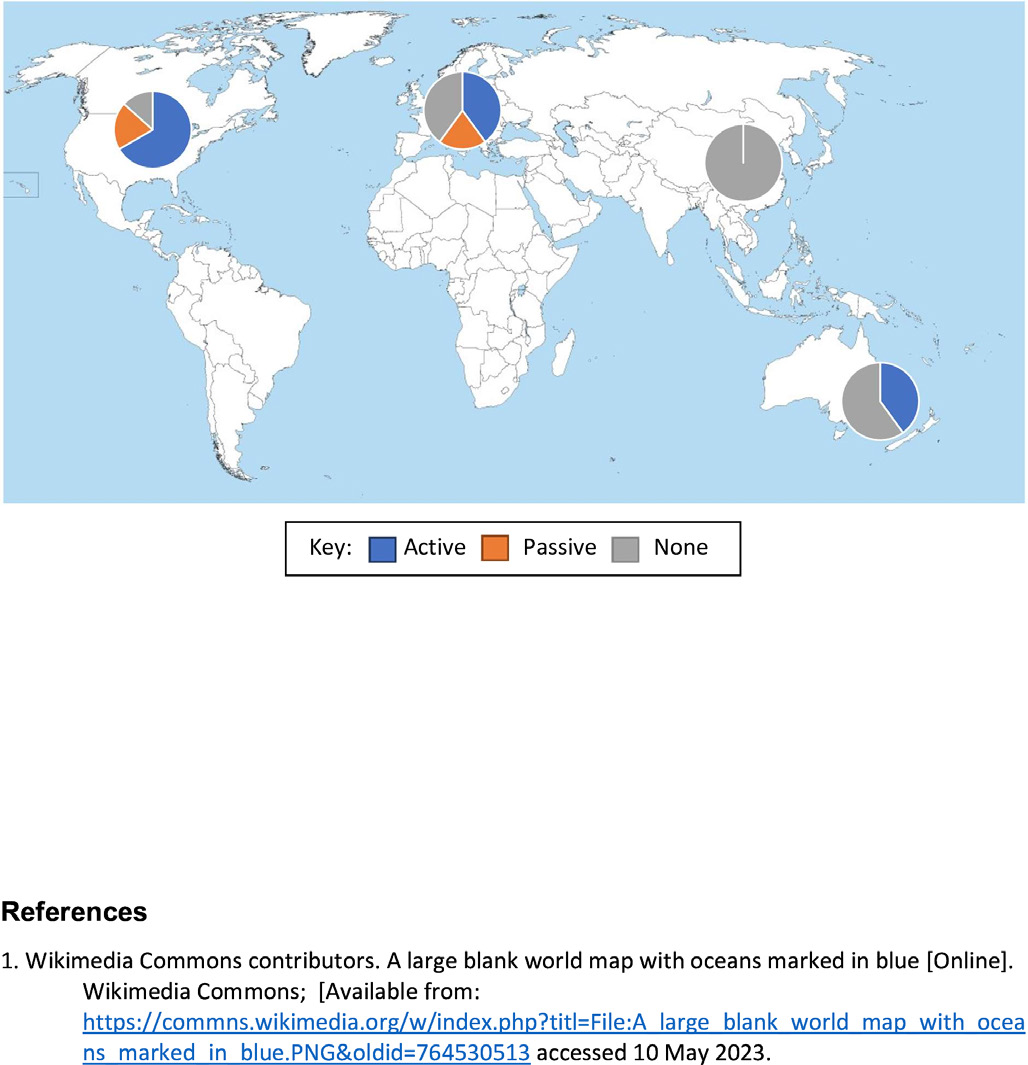
Approach taken to information relatives by continent.

The relative proportions of each approach and the global variation of the approach taken are shown in figure 3. None of the Asian studies provided information to relatives. No information provision is the predominant approach in Australasian studies whereas an active approach is favoured in North America.

**Figure 4:**
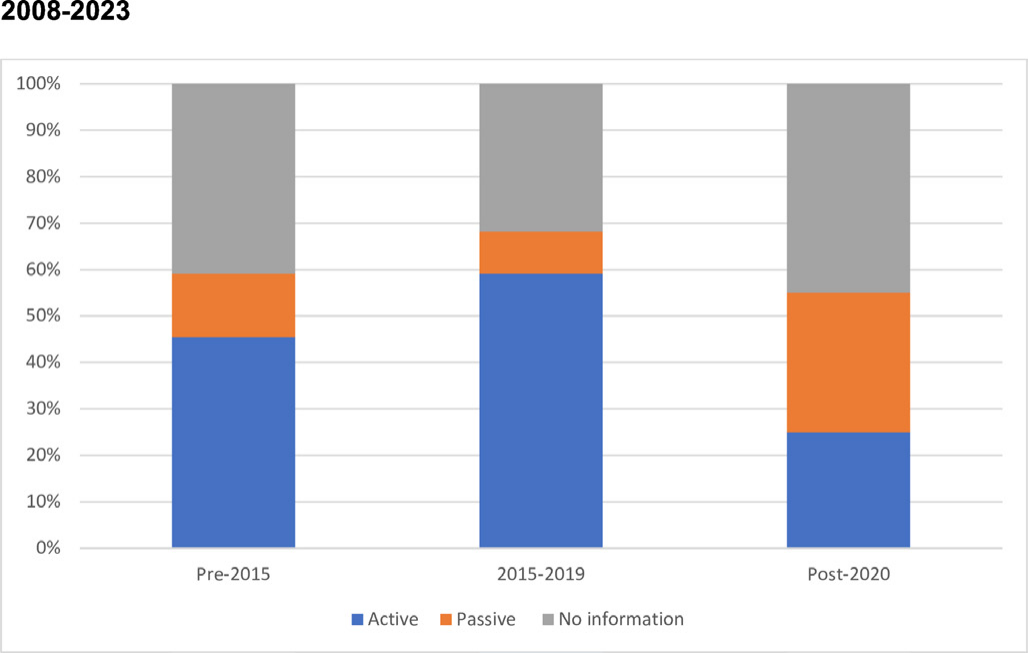
Approach taken to informing relatives in studies opened between 2008 and 2023.

Figure 4 shows how the approach has varied according to the date that trials opened for recruitment. No particular approach has consistently predominated. The reduction in the active approach seen post-pandemic is not related to the continent of origin of the studies.

Of the 64 studies, 23% (n=15) used a model of a community consent whereby researchers engaged with the local community prior to data collection and the community supported the trial. This activity appeared to be independent of the method of informing relatives.

### Method of active approach to informing relatives

Of the 28 studies where relatives were actively informed of the patients’ inclusion, 27 respondents indicated how first contact was made with relatives (figure 2). Of those who responded, this was most frequently by letter or email after the event (n=11). The least frequently used contact strategy was a personal visit by a researcher after the event (n=2). 26 researchers submitted responses regarding the timing of information provision. This ranged from immediately following the resuscitation attempt to 90 days post event. The most frequent timing was within 24 hours; 15 studies delivered information during this time. These studies were mostly European (n=13) but none were recruited in the UK. In four studies at least a month elapsed before relatives were informed. The timing was protocolised in three studies (4–6 weeks in one study, 2 months in two others) to give relatives sufficient time to recover from the immediate grieving period.^11 23 24^ The majority of studies (n=8) opting to delay informing relatives beyond 24 hours recruited patients from North America or Australia.

### Method of passive approach to informing relatives

In 11 studies researchers took a passive approach to informing relatives. In these cases, general information about the study and contact details for the research team were shared via public channels. This was designed to enable relatives to decide whether to seek further information about the study such as whether their relative was enrolled. Most studies shared information via more than one medium. The most popular means of sharing information was via posters placed in community locations likely to be frequented by the relatives of non-surviving patients, such as emergency department waiting rooms and register offices. This method was cited in 10 studies. Except for one study opening in 2011, most of the studies using electronic media opened more recently (2018–2023).

### What influenced choice of approach

Researchers revealed that there were many different influences on their choice of approach to informing relatives (table 2). Of the 62 studies where an influence was reported, 45 indicated it was a requirement of the Research Ethics Committee (REC). However, this was not the dominant influence across all studies. Previous research experience (n=8) and advice from public groups (n=7) were more frequently indicated as determinants of the adoption of a passive approach. Those studies using a ‘no information’ approach were most strongly influenced by the REC.

**Table 2:** Influences on approach to informing relatives of non-survivors

	Active (n=28)	Passive (n=11)	No info (n=64)	All approaches (n=64)
Requirement of the Research Ethics Committee	74% (n=20)	55% (n=6)	79% (n=19)	73% (n=45)
Previous research experience	59% (n=16)	73% (n=8)	29% (n=7)	48% (n=30)
Legal requirement	37% (n=10)	9% (n=1)	46% (n=11)	36% (n=22)
Advice from other researchers	30% (n=8)	55% (n=6)	17% (n=4)	29% (n=18)
Advice from public groups	15% (n=4)	64% (n=7)	21% (n=5)	26% (n=16)
Societal expectation	11% (n=3)	18% (n=2)	17% (n=4)	15% (n=9)
Other: cultural/situational considerations	4% (n=1)	0%	0%	2% (n=1)
Other: prevention of related bias	4% (n=1)	0%	0%	2% (n=1)
Other: feeling that relatives should be informed	7% (n=2)	0%	0%	2% (n=1)
N/A	0%	0%	0%	3% (n=2)
Did not answer	4% (n=1)	0%	4% (n=1)	3% (n=2)

When asked for their *rationale* for opting to provide no information to relatives, researchers often cited more than one reason (figure 2). Most often it was simply that there was no requirement to inform relatives (n=16). Researchers commonly indicated that *they* felt it would be inappropriate to inform relatives that their loved one had been enrolled in a research study (n=12).

### Concerns/issues resulting from chosen approach

The investigators who used an active approach to informing relatives did not report any issues/concerns from relatives (figure 2). In one study, relatives contacted researchers to say that they viewed the study enrolment positively and, in another, relatives had got in touch to find out whether any action was required on their part. In the 10 studies that had recruited participants, no issues were encountered with a passive approach. In the majority (n=20) of studies where no information was provided, investigators reported no concerns. In four studies, researchers reported concerns/issues arising from the approach taken. These included two studies where issues were received from relatives and a further two studies where relatives had misunderstandings about death. This question was not answered in one study.

## Discussion

In this systematic analysis of the approach taken to informing the relatives of non-surviving patients enrolled in cardiac arrest studies we found substantial variability in practice. In the last 15 years, of the studies where there was a response, 28 studies actively informed relatives, 25 provided no information and in 11 studies information was provided passively. The most common means of active contact was via letter or email. The most frequent timing for notification was within the first 24 hours post-mortem, although the maximum delay was 90 days before delivering this news. We are moderately confident that our findings are generalisable as we obtained information on 62% of the target population and the sample was diverse.

RECs commonly influenced the approach taken. However, it is not clear whether it was the REC or the local legal/regulatory requirements that were the key determinants. In Australia, legal requirements vary by jurisdiction. In some states there is no legal precedent for enrolling patients with impaired consent in research, though it is recognised that such enrolments do occur.^25^ Even where legal processes do exist, there is no specific requirement of informing relatives of non-survivors.^25^

In the UK patients may be enrolled in emergency medicines trials without prior informed consent under the provisions of the Medicines for Human Use (Clinical Trials) (Amendment) Regulations (2019) or other research in England and Wales under the Mental Capacity Act (2005).^26 27^ Neither of these give explicit guidance on whether to advise the bereaved family of their relative’s enrolment.^28^ In the absence of legal guidance, practice has been found to vary. Some researchers reported routinely seeking permission for the use of data from family members; others did not since a valid professional legal consultee declaration negated the need to put relatives through additional distress.^28^ In Scotland, patients may be enrolled in medicines trials, but the Adults With Incapacity (Scotland) Act 2000 does not make provision for patients to be enrolled in other types of research.^29^ Although a Scottish REC may consider such research, there would likely be subtle differences in practice within a UK-wide study.

The variability in approach reflects the fact that only 36% of studies’ approaches were mandated in law. It is also likely to be influenced by the prevailing culture. For example, talking about death is considered disrespectful or blasphemous in Chinese culture.^30^ In this context, actively informing relatives about research participation would likely be extremely uncomfortable for both researchers and relatives. This may help to explain the consistent ‘no information’ approach we found in the Asian studies. Where researchers do not actively inform relatives, there is a risk that they may find out through ‘uncontrolled’ channels such as the media. Disclosure through such channels may lead to inaccuracies.^31^ Where researchers used a passive strategy, this was most frequently via posters in targeted locations such as the waiting rooms of primary care physicians. However, variable effectiveness of this strategy has been reported, with between 5% and 78% of patients noticing posters in the waiting rooms of General Practitioner (GP) practices or hospital emergency departments.^32 33^

A widespread passive provision of trial information may require significant research funding, with no guarantee that the information has reached the relevant target audience.^32^ Active direct notification by writing to family members may be more specific but researchers have warned that the process is cumbersome, costly and resource-intensive.^8^ Additionally, this precise targeting is limited by the ability of researchers to reliably obtain contact information for relatives. Researchers using postal notification have previously reported finding either incorrect or no contact details for relatives in 9–18% of cases.^8 9^ Timing of the notification also needs careful consideration. We found that most studies contacted relatives within 24 hours of death. This contrasts with previous studies which report median notification delivery times of 6–8 days.^9 10^ There is a risk that high withdrawal rates following notification of research participation may bias the research findings. In practice, this rarely occurs; between 0% and 0.91% of withdrawals have followed active notifications.^8^,^10^

Our study has several important limitations. First, we may have increased our response rate had we followed-up by phone as well as email, although this was precluded by resource constraints. Second, we observed some differences between trials that responded and trials that did not respond, which may reflect some selection bias. For example, most of our responses were from Europe which may lead to a cultural bias in the findings. Third, some researchers represented multiple studies (a cluster) and the approach in these studies was usually homogeneous. This clustering effect may have introduced bias, as if you respond for one study you are more likely to respond for another. Finally, we did not ask whether regulations regarding emergency research had changed during the period of interest.

The most important perspective missing from the literature at this time is the perspective of the recipient of the notifications in whichever form. Qualitative research exploring their experiences would be an important contribution to the literature. This may also form a precursor to subsequent work looking to standardise the approach.

In summary, we found wide variability in the approach taken to informing relatives of non-surviving patients enrolled in cardiac arrest studies and researchers cited a variety of influences on their selection of approach.

## Supplementary material

10.1136/emermed-2023-213648 online supplemental file 1

## Data Availability

Data sharing not applicable as no data sets generated and/or analysed for this study.
