# Supplementary material for: Systematic analysis of approaches used in cardiac arrest trials to inform relatives about trial enrolment of non-surviving patients
Source: Emerg Med J. 2024 May 10;42(8):e213648. doi: 10.1136/emermed-2023-213648 (PMC12322478; doi:10.1136/emermed-2023-213648)
Supplement: online supplemental file 1 [file emermed-42-8-s001.pdf]

Supplemental material

Registry search strategies

The following strategies were employed:

- *ClinicalTrials.gov*

  TI: “Cardiac arrest”

  First posted: 01/01/2007 – 10/04/2022 (US format, as per registry)

  Study type: Interventional

- *WHO ICTRP*

  “Cardiac arrest”

  Recruitment status: All

  Date of registration: 01/01/2007 – 04/10/2022

  Study type: Interventional

Data collection tool

Q1: Did your trial enrol participants without prior informed consent (e.g., waiver of consent/ deferred consent)?

☐ Yes (but used a model of community consent whereby engagement with the local community showed community approval for this approach)

☐ *Yes, but did **not** use community consent model.*

☐ *No*

For the following questions, please base your responses on the country/ region where most participants were recruited (you will be asked to clarify differences across regions at the end of the survey).

Q2: Where a participant was enrolled in the trial without consent and subsequently died before they or their relative could be informed about the trial, what method did you use for approaching their relative? (Please choose closest option)

☐ Active information (I.e. you made a specific approach (e.g. by letter/ phone call/ visit) to inform them about trial participation)

☐ Passive information (I.e. information about the trial was placed in the public domain that allowed the relative to contact the trial team if they wanted further information)

☐ No information

Q3: Where a participant was enrolled in the trial without consent and subsequently died before they or their relative could be informed about the trial, what method did you use for approaching their relative? (Please choose closest option)

☐ Active information (I.e. you made a specific approach (e.g. by letter/ phone call/ visit) to inform them about trial participation)

☐ Passive information (I.e. information about the trial was placed in the public domain that allowed the relative to contact the trial team if they wanted further information)

☐ No information

(If **ACTIVE** approach taken......)

a. How was the first contact made?

☐ Information by clinician at the scene/ emergency department

☐ Email/ letter

☐ Phone call

☐ Personal visit by researcher/ clinician after the event

b. What was the purpose of this approach?

☐ Information only

☐ Seek consent for specific aspect of trial (e.g. use of data)

(If approached via email/ letter, phone call, or personal visit....)

c. When was this contact usually made?

(If **PASSIVE** approach taken ….)

How did you communicate this information in the public domain?

- [ ] Posters located in hospitals/register office/funeral directors/community location
- [ ] Leaflets located in community locations
- [ ] Social Media Marketing
- [ ] Radio / Newspaper
- [ ] Other – Please state

(If **NO INFORMATION** provided...)

Why did you make this decision?

- [ ] No requirement to inform relatives
- [ ] We felt it would be inappropriate to inform relatives
- [ ] Our community consultation advised us against informing relatives
- [ ] Other

Q4: What factors influenced your approach to informing relatives of participants that did not survive about trial participation?

- [ ] Legal requirement
- [ ] Requirement of ethics committee
- [ ] Advice from members of the public/ community groups
- [ ] Previous research experience
- [ ] Advice from other researchers
- [ ] Societal expectation
- [ ] Other - please state

Q5: Did your chosen approach to informing relatives of participants that did not survive about trial participation create any specific issues?

- [ ] Negative media reports
- [ ] Complaints from relatives
- [ ] Complaints from community groups
- [ ] Criticism from clinical colleagues/ other researchers
- [ ] Other- please state.

Q6 To what extent do you agree with the following statement:

“The approach that we used for informing next of kin of non-surviving trial participants was the correct approach”.

Strongly disagree	Disagree	Neutral	Agree	Strongly agree
☐	☐	☐	☐	☐

Q7 **-** Do you wish to provide any additional information? (e.g. if your trial was multi-national, did your approach vary across countries)

Numerical data from pie charts, Figure 2

	Active (n = 28)	Passive (n = 11)	No info (n = 25)
No issues	23	10	20
Have not yet implemented approach	0	1	0
Some relatives viewed the study enrolment positively	1	0	0
Some relatives asked if there was anything they needed to do	1	0	0
Caused misunderstanding about death	0	0	0
Complaints from relatives	0	0	2
Did not answer	3	0	2

Characteristics of included registry study records

Trial name	Date of registration	Recruitment period	Country of recruitment	Continent	Population	Intervention	Comparator	Primary Outcome
SB CAT	16/06/2011	2011-2013	Israel	Asia	Adults with non-traumatic OHCA with established vascular access	Sodium bicarbonate following first dose adrenaline	Saline placebo following first dose adrenaline	ROSC and Survival to hospital arrival
EXACT	21/04/2017	2017-2020	Australia	Australasia	Adults with OHCA	Post ROSC oxygen titrated to maintain SpO2 between 90-94%	Post ROSC oxygen titrated to maintain SpO2 between 98-100%	Survival to hospital discharge
Feasibility study of normoxic versus hyperoxic therapy after cardiac arrest	03/10/2012	2012-2013	New Zealand	Australasia	Adults with ROSC with advanced airway following OHCA with initially shockable rhythm	Titrated oxygen to target 90-94%	Standard care (100% oxygen)	Mean oxygen saturation by minute in prehospital phase of care
A study of patients in cardiac arrest due to ventricular arrhythmias to determine if manual pressure augmentation during defibrillation improves neurological outcome and survival	25/06/2021	2021-2024	Australia	Australasia	Adults with OHCA with initially shockable rhythm	200J shocks with manual pressure on defibrillation pads	200J with no manual pressure	Survival to hospital discharge
RINSE	26/07/2010	2010-2014	Australia	Australasia	Adults with OHCA	Paramedic-initiated cooling	Standard care	Survival at hospital discharge

A study of two existing transfer options: expedited transfer versus non-expedited transfer from scene to hospital in of out of hospital cardiac arrest patients treated and the impact on survival.	01/06/2021	2021-2023	Australia	Australasia	Adults with witnessed OHCA with initially shockable rhythm receiving bystander CPR within <5 minutes	Expedited transport to ECMO centre with mechanical CPR	Usual care	Survival with favourable neurological outcome (CPC1-2) at discharge
SAVE	18/11/2016	2016-2019	Taiwan	East Asia	Adults (over 20yrs) with OHCA receiving advanced airway device	Endotracheal tube	Supraglottic airway device	Survival at 1 month
VICTOR	16/10/2019	2020-2022	Taiwan	East Asia	Adults with non-traumatic OHCA	IO vascular access at humeral head site	IV vascular access	Survival to hospital discharge
Effectiveness of chest compression for patients with out-of-hospital cardiac arrest	01/06/2012	2012-unknown	Japan	East Asia	Adults over 20 years with bystander witnessed OHCA	CPR with perfusion index (RAD-57) monitoring	Standard CPR	Frequency of ROSC
Effect of feedback by NIRO-CCR1 during cardiopulmonary resuscitation for patients with out-of-hospital cardiac arrest	28/06/2015	2015-unknown	Japan	East Asia	Adults with non-traumatic OHCA	Monitoring by NIRO-CCR1 during CPR	Standard CPR without monitoring	Rate of ROSC

Intraosseous Versus Intravenous Vascular Access During Resuscitation Following Out-of-Hospital Cardiac Arrest	16/10/2019	2020-2022	China	East Asia	Adults with OHCA	IO vascular access	IV vascular access	ROSC within 24 hours
Cardiac Arrest and Ventilation Method	22/09/2022	2021-2022	Republic of Korea	East Asia	Adults with OHCA receiving at least 20min ACLS in the ED	Automatic mechanical ventilation	Manual ventilation	ROSC within 20min of ACLS
CORTICA	24/05/2016	2016-2018	Greece	Europe	Adults with IHCA	Steroids during CPR	Saline placebo during CPR	Arterial pressure and central venous oxygen saturation 72hrs post ROSC
AMSA	21/07/2017	2019-2021	Italy	Europe	Adults with OHCA with shockable rhythm	AMSA guided CPR	Standard CPR	ROSC within 1hr
GRAVITY	17/06/2019	2019-2022	France	Europe	Adults with cardiac arrest	Head up position, impedance threshold device & automated CPR	Standard CPR	Maximum $EtCO_2$ on day 0
COCA	03/11/2019	2020-2021	Denmark	Europe	Adults with OHCA	Calcium chloride	Sodium chloride	ROSC within 2hrs of cardiac arrest
PRINCESS	22/07/2011	2010-2018	Belgium, Sweden	Europe	Adults with OHCA	Prehospital intra-nasal cooling with RhinoChill	Standard ACLS with hypothermia according to local protocol	Neurologically intact survival (CPC1-2) at 90 days
TANGO2	30/03/2015	2017-2022	Sweden	Europe	Adults with OHCA	Compression-only CPR with continuous compressions	Standard CPR with rescue breaths (30:2)	Feasibility and safety of study assessed at 6 months
EMERGE	23/08/2016	2017-2020	France	Europe	Adults with ROSC following OHCA	Delayed coronary angiography	Immediate coronary angiography	Survival with favourable neurological outcome (CPC1-2) at 6 months

INCEPTION	05/04/2017	2017-2021	Netherlands	Europe	Adults with OHCA with refractory shockable rhythm	ECPR	Standard ACLS	Survival with favourable neurological outcome (CPC1-2) at 30 days
VAM-IHCA	21/08/2018	2018-2021	Denmark	Europe	Adults with IHCA	Methylprednisolone + vasopressin following adrenaline administration	Saline following adrenaline administration	Survival to 30 days and Favourable neurological outcome at 30 days
CYTER	26/09/2018	2019-2020	Germany	Europe	Adults admitted to ITU following successful ECPR	Cytosorb removal column in ECPR	Standard ECPR	Survival at 30 days
Prehospital Non-invasive Cooling of Comatose Patients After Cardiac Arrest	28/01/2019	2021-2022	Austria	Europe	Adults with ROSC following witnessed cardiac arrest	Prehospital cooling with CAREvest device	No prehospital cooling	Cooling rate within 4 hours
PHTEE	28/04/2022	2022-unknown	Germany	Europe	Adults with OHCA	Transoesophageal echocardiography guided resuscitation	Standard care	Hands-off times
STEROHCA	30/03/2020	2020-2022	Denmark	Europe	Adults with OHCA of presumed cardiac cause with ROSC for ≥ 5 minutes	Methylprednisolone + vasopressin following adrenaline administration	Saline placebo following ROSC	Concentration of interleukin 6 and neuron-specific enolase at 72 hours post admission
AIRWAYS2	28/07/2014	2015-2018	United Kingdom	Europe	Adults with non-traumatic OHCA	Supraglottic airway device used in ventilation during OHCA	Endotracheal ventilation during OHCA	Survival and functional neurological outcome (mRS) at discharge
AIRWAYS3	29/07/2022	2022-2026	United Kingdom	Europe	Adults with IHCA requiring advanced airway management	Supraglottic airway device used in ventilation during IHCA	Endotracheal ventilation during IHCA	Survival and functional neurological outcome (mRS) at discharge
VANZ-2	18/08/2022	2021-2022	United Kingdom	Europe	Adults with OHCA	Real-time ventilation feedback	Standard care without feedback	Compliance with ERC guidelines for ventilation
NICA	08/04/2019	2019-2022	Germany	Europe	Adults with cardiac arrest	Modified CPR protocol according to cerebral oximetry readings	Standard CPR protocol	Rate of ROSC
IVIO	11/01/2022	2022-2024	Denmark	Europe	Adults with OHCA requiring vascular access	IO vascular access	IV vascular access	Sustained ROSC (>20min)

ARREST	13/03/2019	2018-2023	United Kingdom	Europe	Adults with ROSC following OHCA of cardiac cause	Expedited transport to cardiac arrest centre	Standard care	All-cause mortality at 30 days
Influence of Morphine or Ketamine or Saline Applied During In-hospital Cardiopulmonary Resuscitation on Early Survival	05/07/2019	2021-2025	Norway	Europe	Adults with IHCA	Morphine or ketamine during CPR	Saline placebo during CPR	Survival at 28 days
REBOARREST	22/10/2020	2021-2024	Norway	Europe	Adults with non-traumatic OHCA with time of arrest to initiation of CPR <10 minutes	Resuscitative Endovascular Balloon occlusion of the aorta (REBOA)	Standard Advanced Cardiovascular Life Support	Sustained ROSC (>20min)
LUCAT	30/01/2012	2012-2014	Spain	Europe	Adults with witnessed non-traumatic OHCA with response time <12 minutes	LUCAS mechanical chest compressions	Manual chest compressions	Survival to hospital admission and to hospital discharge
PARAMEDIC 2	19/03/2014	2014-2017	United Kingdom	Europe	Adults with OHCA	Adrenaline	Saline placebo	Survival to 30 days
REVIVE-Airways	24/05/2012	2012-2013	United Kingdom	Europe	Adults with OHCA	iGel airway device	Standard care	Feasibility of full scale trial
COMPRESS-RCT	12/01/2017	2017-2019	United Kingdom	Europe	Adults with IHCA in a non-shockable rhythm	LUCAS mechanical chest compressions	Manual chest compressions	Proportion of eligible patients randomised during operational recruitment hours
PARAMEDIC-3	16/08/2021	2021-2023	United Kingdom	Europe	Adults with OHCA requiring vascular access	IO first strategy	IV first strategy	Survival to 30 days
POSED	23/06/2021	2022-2023	United Kingdom	Europe	Adults with OHCA requiring shock	150-200-200J/ 200-200-200J	120-150-200J	Recruitment rate
PARAMEDIC	10/02/2009	2009-2013	United Kingdom	Europe	Adults with OHCA	LUCAS mechanical chest compressions	Manual chest compressions	Survival to hospital discharge

SAFETY study	11/09/2009	2009-2014	Netherlands	Europe	Adults with OHCA	Autopulse mechanical chest compressions	Manual chest compressions with audio-visual feedback	CPR-related damage
SAFETY study	11/09/2009	2009-2014	Netherlands	Europe	Adults with OHCA	LUCAS mechanical chest compressions	Manual chest compressions with audio-visual feedback	CPR-related damage
PROXY	24/11/2016	2014-2015	United Kingdom	Europe	Adults with non-traumatic OHCA achieving ROSC for 2 minutes	100% oxygen	Oxygen titrated according to patient's oxygen levels	Proportion of eligible paramedics trained and consenting to take part in the trial
ON-SCENE	26/10/2020	2021-2025	Netherlands	Europe	Adults ≤50 years with refractory witnessed OHCA with initial rhythm of VF/VT or suspected pulmonary embolism	Physician managed resuscitation with option of prehospital ECPR	Standard paramedic managed resuscitation without prehospital ECPR	Survival to hospital discharge with favourable neurological outcome (CPC1-2) and costs related to ECPR per QALY
COMACARE	04/03/2016	2016-2017	Finland	Europe	Adults with ROSC following OHCA with initially shockable rhythm	8 comparator groups varying high/low/normal PaO_2, $PaCO_2$ and MAP for 36hrs	Factorial design	Neuron-specific enolase serum concentration at 48hrs post arrest
VSE-2	08/08/2008	2008-2010	Greece	Europe	Adults with IHCA requiring epinephrine	Vasopressin, epinephrine, and methylprednisolone during CPR; hydrocortisone after CPR	Standard ALS	ROSC for ≥15 minutes and survival to hospital discharge
HITUPPAC-BIO	22/04/2009	2009-2012	France	Europe	Adults with non-traumatic OHCA	Prehospital hypothermia	Hypothermia at hospital	Reduction of brain damage biomarkers at 72hrs
CILICA-HS	24/01/2019	2019-2024	France	Europe	Adults with cardiac arrest	CPRmeter© feedback device	CPR without feedback	Chest compression fraction and correct compression score
The Haemodynamic Effects of Mechanical Standard and Active Chest Compression-decompression During Out-of-hospital CPR	24/06/2015	2015-2017	Norway	Europe	Adults with non-traumatic OHCA	Chest compressions with LUCAS2AD (active decompression above the initial position of the suction cup)	Chest compressions with LUCAS2 device	$EtCO_2$ during 30 minutes of CPR
A pilot study to investigate the use of an	08/11/2018	2013-2017	United Kingdom	Europe	Adults with OHCA of presumed cardiac cause	Impedance threshold device	Standard care	Recruitment rate and treatment compliance

impedance threshold device (ITD) – the ResQPOD, to improve circulation during cardiopulmonary resuscitation (CPR) for patients in cardiac arrest								
Epo-ACR-02	22/10/2009	2009-2013	France	Europe	Witnessed OHCA of cardiac aetiology with ROSC within 60 minutes of arrest	Epoetine alpha	Usual care	Proportion of patients with CPC = 1
APACAR2	13/08/2015	2016-2019	France	Europe	Adults in cardiac arrest with refractory shockable rhythm	ECMO initiated in pre-hospital setting	ECMO initiated in hospital setting	Survival with good neurological outcome (CPC1-2) at 6 months
CYRUS	10/05/2012	2010-2013	France	Europe	Adults with non-shockable OHCA	Cyclosporine A at the onset of resuscitation	Usual care	Sequential Organ Failure Assessment (SOFA) score at 24hrs post admission
The efficacy of amiodarone compared to the efficacy of adrenaline for the treatment of cardiac arrest.	21/02/2014	Unknown	Denmark	Europe	Adults with OHCA with initial rhythm of VF	High dose amiodarone	Adrenaline + low dose amiodarone	Survival to hospital discharge
Landiolol for improved outcome in cardiac arrest	31/08/2020	2021-2022	Austria	Europe	Adults with OHCA with initially shockable rhythm	Landiolol in addition to standard care	Standard care with saline placebo	Time to sustained ROSC
Beta-Arrest	26/09/2022	2021-2022	Austria	Europe	Adults with OHCA with refractory shockable rhythm	Landiolol	Saline	Time to sustained ROSC

Prehospital Laryngeal Tube vs. Bag-Valve Mask Ventilation Used by Paramedics During CPR	31/10/2012	2012-2015	Austria	Europe	Adults with OHCA	Laryngeal tube ventilation	Bag valve mask ventilation	Effective ventilation during the period of out-of-hospital resuscitation
I-CAN	29/01/2014	2014-2016	Norway	Europe	Adults with OHCA	iGel airway device	Standard airway management	Ventilation success (visible chest movement, audible air passage on ventilation, $EtCO_2$ confirmation)
Hyperinvasive Approach in Cardiac Arrest	13/01/2012	2013-2020	Czechia	Europe	Adults with non-traumatic OHCA	Prehospital mechanical chest compressions, intra-arrest cooling and in-hospital ECLS	Standard care	Survival with good neurological outcome (CPC1-2) at 6 months
ORI-ONE	17/07/2018	2018-2022	Belgium	Europe	Adults with ROSC following non-traumatic OHCA	Oxygen titrated according to ORI index + oxygen saturation	Oxygen titrated according to oxygen saturation	Normoxia index at hospital admission
HemOpt-PVI	15/02/2019	2019-2022	Belgium	Europe	Adults with ROSC, lactate >4 mmol/l and MAP below 65mmHg following OHCA	Goal-directed therapy using pleth variability index	Standard non-invasive monitoring	Change of lactate and fluid balance in first 24 hours following ED admission, fluid balance and normalisation of lactate at 48 hours
VICA	01/12/2020	2019-2021	Austria	Europe	Adults with OHCA and established endotracheal intubation	Ventilation performed at 20 breaths per minute	Ventilation performed at 10 breaths per minute	Minute ventilation and adequacy of ventilation (pH and $paCO_2$)
BABICA	27/04/2011	2011-2013	Austria	Europe	Adults with cardiac arrest	Sodium bicarbonate	Sodium chloride	ROSC within 5 hrs of start of resuscitation
VAST-A	17/11/2021	2021-2027	Sweden	Europe	Adults ≤50 years with IHCA requiring adrenaline	Vasopressin and steroids following adrenaline administration	Saline placebo following adrenaline administration	Survival at 30 days
PERSEUS-PS	04/06/2020	2020-2022	Greece	Europe	Adults with established invasive arterial BP monitoring prior to and during IHCA	CPR according to PERSEUS protocol	Standard CPR	ROSC within 1 hour
EP-PCEH	13/09/2013	2013-2016	Spain	Europe	Adults with OHCA	Passive leg raise during CPR	Legs flat during CPR	Survival with favourable neurological outcome (CPC1-2) at hospital discharge

CAAM	23/12/2014	2014-2017	France	Europe	Adults with OHCA	Bag-valve-mask ventilation during OHCA	Endotracheal ventilation during OHCA	Survival with favourable neurological outcome (CPC1-2) at 28 days
ECPB4OHCA	22/05/2012	2014-2020	Austria	Europe	Adults with cardiac arrest	Emergency cardiopulmonary bypass under ongoing CPR	Standard ACLS	ROSC within 2-48hrs
Add-on Cangrelor in STEMI-triggered Cardiac Arrest	06/09/2017	2017-2021	Austria	Europe	Adults with ROSC and STEMI following OHCA with initially shockable rhythm, treated with TTM	Parenteral cangrelor added to standard antiplatelet treatment	Placebo added to standard antiplatelet treatment	Platelet reactivity at stent placement
CYRUS II	18/08/2016	2017-2019	France	Europe	Adults with OHCA with shockable rhythm	Single bolus cyclosporine A at the onset of resuscitation	Single bolus placebo at the onset of resuscitation	Combined incidence of all-cause mortality and irreversible brain damage status at 7 days
Prehospital Intubation of COVID-19 Patient With Personal Protective Equipment	24/04/2020	2020-2020	Poland	Europe	Adult patients with OHCA requiring out-of-hospital intubation	Vie Scope laryngoscopy	Direct laryngoscopy	Intubation success rate during first laryngoscopy
LATTE	30/07/2021	2022-2024	Belgium	Europe	Adults with OHCA with time to sustained ROSC >15 minutes	Infusion with sodium lactate	Standard care infusion	NSE serum levels 48 hours post randomisation
Intraosseous Versus Intravenous Vascular Access During Cardiac Arrest	06/05/2010	2010-2010	United States	North America	Adult patients with a cardiac arrest of medical aetiology	Intraosseous access via 1. humeral head or 2. tibia	Peripheral intravenous access	First attempt access success rate
Pilot Study of Sodium Nitrite in Resuscitated Cardiac Arrest Patients	06/08/2010	2010-2016	United States	North America	Adult patients undergoing resuscitation for OHCA	Sodium nitrite infusion during resuscitation	Normal saline infusion	Blood pressure and nitrite concentration levels in blood at 2hrs
ALPS	08/07/2011	2012-2015	United States; Canada	North America	Adult patients with OHCA with initially shockable rhythm	Amiodarone or lidocaine for VF/pVT post 1st and 2nd shocks	Normal saline post 1st and 2nd shocks	Survival to hospital discharge

Initiation of Cooling by EMS to Promote Adoption of In-hospital Hypothermia in Cardiac Arrest Survivors	31/10/2011	2012-2016	Canada	North America	Adults with OHCA	Prehospital cooling	Standard care	Successful cooling (achievement of target temperature within 6hrs of ED arrival)
EROCA	16/02/2017	2017-2020	United States	North America	Adults with cardiac arrest	Expedited transport to ECMO centre with mechanical CPR	Standard ACLS with standard CPR	ED arrivals and ECPR within 30 min
DOSEVF	06/09/2019	2019-2022	Canada	North America	Adults with non-traumatic OHCA and refractory shockable rhythm after 3 shocks	Vector change defibrillation/ Double sequential defibrillation	Standard defibrillation	Survival at hospital discharge
The Application of Ketamine for Sedation in Patients With Cardiac Arrest	21/04/2020	2022-2023	Canada	North America	Adult patients with ROSC requiring sedation following OHCA with shockable rhythm	Ketamine hydrochloride for sedation	Sedation without ketamine hydrochloride	Feasibility of study assessed at 6 months
HART	30/08/2022	2023-2027	United States	North America	Adults with IHCA	First choice supraglottic airway device	First choice intubation	Alive and ventilator-free days within 28 days
SNOCAT	12/02/2018	2018-2019	United States	North America	Adults with OHCA with established vascular access	Sodium nitrite (45mg or 60mg)	Saline placebo	ROSC at hospital arrival
EpiDOSE	01/02/1019	2021-2025	Canada	North America	Adults with OHCA with initially shockable rhythm and established vascular access	Low dose epinephrine	Standard dose epinephrine	Survival to hospital discharge
ReTEECA	07/01/2020	2022-2024	United States	North America	Adults with IHCA and advanced airway in situ	ACLS guided by Transtracheal echocardiography	Standard ACLS	Survival to hospital discharge
EPR-CAT	05/01/2010	2016-2023	United States	North America	Cardiac arrest following traumatic OHCA	EPR protocol: rapid induction of hypothermia, resuscitative surgery with cardiopulmonary bypass	Standard resuscitation	Survival to hospital discharge without major disability (Glasgow Outcome Scale-Extended>5)

Goal-directed CPR Using Cerebral Oximetry	10/06/2021	2022-2025	United States	North America	Adults with IHCA	Physiological feedback CPR	Non-physiological feedback CPR	ROSC at day 0
Hemodynamic Effects of Standard Cardiopulmonary Resuscitation (CPR), Active Compression Decompression CPR With an Inspiratory Impedance Device, and Standard CPR With an Intrathoracic Pressure Regulator During Out-of-hospital Cardiac Arrest	28/03/2011	2011-2012	United States	North America	Adult patients with non-traumatic OHCA	Active compression decompression CPR with ResQPRO device and ResQPOD ITD device	Standard manual CPR	Mean systolic and diastolic blood pressures during CPR and SAEs during CPR, at hospital discharge, 30 days, 3 months and 6 months
CIRC	17/01/2008	2008-2011	United States	North America	Adults with non-traumatic OHCA	Mechanical CPR with Autopulse	Manual CPR	Survival to discharge
Therapeutic Hypothermia After the Return of Spontaneous Circulation	12/06/2012	2013-2014	United States	North America	Adults with ROSC following non-traumatic OHCA	Induced therapeutic hypothermia	Standard care	Improved outcomes at hospital discharge
A Pilot Study of Intra-arrest Therapeutic Hypothermia in Patients Suffering Non-Traumatic Out of Hospital	09/08/2011	2011-2012	United States	North America	Adults with non-traumatic OHCA	Intra-arrest hypothermia	Standard care	Survival to hospital discharge

Cardiac Arrest								
PART	25/02/2015	2015-2017	United States	North America	Adults with non-traumatic OHCA	Airway management with endotracheal tube	Standard airway management	Survival at 72 hours post arrest
CCC	02/06/2011	2011-2025	United States; Canada	North America	Adult patients with OHCA receiving chest compressions	Continuous compressions	Standard compressions	Survival to hospital discharge
RICE pilot	12/02/2020	2020-2020	United States	North America	Adults with ROSC following non-traumatic OHCA	Remote ischaemic conditioning	Sham ischaemic conditioning	Attrition at 30 min post intervention
ARREST	11/03/2019	2019-2020	United States	North America	Adults with OHCA with shockable rhythm	ECMO	Standard ACLS	Survival to hospital discharge
SPEAR	17/03/2022	2022-2027	United States	North America	Adults with OHCA with PEA	Half normal saline with calcium chloride	Normal saline with calcium chloride	ROSC at hospital arrival
UP-FRONT	11/02/2022	2022-2023	United States	North America	Adults with witnessed OHCA with shockable rhythm	REBOA	Standard care	Time to successful deployment of REBOA device
CAPITALCHILL	08/11/2013	2013-2020	Canada	North America	Adults with cardiac arrest	Therapeutic hypothermia at 31°:C	Therapeutic hypothermia at 34°C	Death or poor neurological outcome (DRS>5) at 6 months
Comparison of manual cardiopulmonary resuscitation with resuscitation using a mechanical device (AutoPulse®) in patients with in-hospital cardiac arrest	25/07/2013	2013-unknown	India	South Asia	Adults aged 18-60yrs with cardiac arrest in the ED	Mechanical chest compressions with Autopulse device	Manual chest compressions	ROSC and survival at 24 hours and to discharge
AMCPR Trial for OHCA	13/06/2017	2017-2021	Republic of Korea	Southeast Asia	Adults with non-traumatic OHCA with non-shockable rhythm	Vasopressin for arterial diastolic blood pressure less than 20 mmHg	Saline for arterial diastolic blood pressure less than 20 mmHg	Sustained ROSC (>20min)

Bicarbonate in Patients With Out-of-hospital Cardiac Arrest	23/11/2014	2014-2016	South Korea	Southeast Asia	Adults with non-traumatic OHCA	50ml Sodium bicarbonate	50ml Saline	Sustained ROSC (>20min)
Effect of Vasopressin, Steroid, and Epinephrine Treatment in Patients With Out-of-hospital Cardiac Arrest	04/10/2017	2018-2020	Republic of Korea	Southeast Asia	Adults with non-traumatic OHCA treated in the ER	Epinephrine + Vasopressin/ Epinephrine + steroid/ Epinephrine + vasopressin + steroid during CPR	Epinephrine during CPR	Survival with favourable neurological outcome (CPC1-2) at discharge
Compare Outcomes of CPR Between the Video-laryngoscopy (VL) Users and the Direct-laryngoscopy (DL) Users	17/08/2017	2011-2016	Republic of Korea	Southeast Asia	Adults with OHCA	Videolaryngoscopy for endotracheal intubation	Direct laryngoscopy for endotracheal intubation	Survival with favourable neurological outcome (CPC1-2) at 6 months
Videolaryngoscopy During CPR for Trauma Patients	17/08/2017	2011-2015	Republic of Korea	Southeast Asia	Adults with OHCA	Videolaryngoscopy for endotracheal intubation	Direct laryngoscopy for endotracheal intubation	Successful insertion of endotracheal tube on first attempt
Ultrasound in cardiac arrest	04/10/2018	2017-2018	Iran	Western Asia	Adults with OHCA with asystole or PEA	Point of care ultrasound to aid diagnosis	Standard care	Detection of organised contractions, cardiac tamponade, or ventricular enlargement during resuscitation
